# Fracture detection from Azimuth-dependent seismic inversion in joint time–frequency domain

**DOI:** 10.1038/s41598-020-80021-w

**Published:** 2021-01-14

**Authors:** Xinpeng Pan, Dazhou Zhang, Pengfei Zhang

**Affiliations:** School of Geoscience and Info-Physics, Central South University, Changsha, 410082 Hunan China

**Keywords:** Solid Earth sciences, Geophysics

## Abstract

Detection of fracture properties can be implemented using azimuth-dependent seismic inversion for optimal model parameters in time or frequency domain. Considering the respective potentials for sensitivities of inversion resolution and anti-noise performance in time and frequency domain, we propose a more robust azimuth-dependent seismic inversion method to achieve fracture detection by combining the Bayesian inference and joint time–frequency-domain inversion theory. Both Cauchy Sparse and low-frequency constraint regularizations are introduced to reduce multi-solvability of model space and improve inversion reliability of model parameters. Synthetic data examples demonstrate that the frequency bandwidth of inversion result is almost the same for time, frequency and joint time–frequency domain inversion in seismic dominant frequency band using the noise-free data, but the frequency bandwidth in joint time–frequency domain is larger than that in time and frequency domains using low- signal-to-noise-ratio (SNR) data. The results of cross-correlation coefficients validate that the joint time–frequency-domain inversion retains both the excellent characteristics of high resolution in frequency-domain inversion and the advantage of strong anti-noise ability in time-domain inversion. A field data example further demonstrates that our proposed inversion approach in joint time–frequency domain may provide a more stable technique for fracture detection in fractured reservoirs.

## Introduction

Naturally occurring high-density fractures can be seen as ‘sweet spots’ of relatively high permeability for hydrocarbon reservoirs, and knowledge of fracture information is required to determine the fluid flow and optimize the hydrocarbon production in oil and gas fractured reservoirs, such as carbonate reservoirs, unconventional tight sand and reservoirs and shale reservoirs^1,2^. Therefore, detection of natural fractures plays a significant role in seismic characterization for fractured reservoirs, while it is a challenging problem to describe the fractures due to the limited data available to fractures.

Different methods of fracture detection via seismic reflected amplitude data have been used to obtain the fracture information of rocks. S-wave (or shear wave) data is more sensitive to the fracture parameters than P-wave (or compressional wave) data, but it is not used on a large scale because of the costly acquisition and processing, and the limited shear-wave sources available^3^. Converted PS-wave data can be generated via a compressional source with less cost and labor intensive than SS-wave data, which exists more information for fracture detection than the PP-wave data^4–7^. However, it is more complicated in acquisition and processing than non-converted PP-wave data. In general, PP-wave data with azimuthal information is still the most widely used data to estimate the fracture parameters^8^. In this paper, we just use the azimuth-dependent PP-wave reflection amplitude data to implement the fracture detection, but the approach proposed in this paper can be easily extended to the SS-wave inversion, converted PS-wave inversion, or joint PP- and PS-wave inversion.

The effect of fractures on the seismic wave propagation can be characterized in terms of the normal and shear fracture weaknesses ($\delta_{N}$ and $\delta_{T}$) of rocks, in which the normal weakness $\delta_{N}$ exhibits dependence on the fluid content filling fractures, while the shear weakness $\delta_{T}$ is only related to fracture density of rocks^9,10^, and their definitions are presented in “Appendix A”. In the case of a set of parallel, vertical and rotationally invariant fractures embedded in a homogenously isotropic background rocks, the normal and shear weaknesses ($\delta_{N}$ and $\delta_{T}$) of fractures can be used to describe the effective elastic stiffness matrix of a transversely isotropic (TI) medium with a horizontal axis of symmetry^11^. Following the relationship between fracture weaknesses and Thomsen’s anisotropic parameters, a weak-anisotropy and linearized PP-wave reflection coefficient can be derived based on the seismic scattering theory and the first-order perturbations in stiffness components of rocks, building the bridge between the microscopic fracture parameters and macroscopic seismic reflection response^12–15^. As a result, the sensitive weaknesses can be estimated by combining the azimuth-dependent PP-wave seismic data and reflection coefficient equation.

Amplitude versus offset and azimuth (AVOA) inversion has been an important method to predict the fracture information via the PP-wave azimuthal seismic data^16^. Gary et al. use the AVOA inversion to estimate the fracture density and fracture strike from the PP-wave seismic data^17^. Bachrach et al. also use the PP-wave seismic data to reconstruct the Thomsen-type anisotropic parameters based on the rock-physic-based Bayesian inversion in time domain^18^. Chen et al. estimate the sensitive fracture weakness parameters based on the difference in PP-wave azimuthal seismic data, and the proposed method is applied to a field data set^19^. Far et al. just use the synthetic data to estimate the sensitive fracture parameters, but they extend the inversion method to an arbitrary anisotropic medium^20^. In addition, Downton and Roure use the azimuthal Fourier coefficients to estimate the fracture weakness parameters^21^. The reflection-coefficient-based or Fourier-coefficient-based AVOA inversion mentioned above is generally performed in time domain. The inversion in time domain behaves better for the noisy seismic data and worse for the resolution of inversion results^22,23^. The seismic inversion in frequency domain has the advantages of high resolution of inversion results, but anti-noise performance is not good. Combining the time-domain seismic inversion, the seismic inversion in joint time–frequency domain can achieve a balance between inversion resolution and anti-noise performance^24,25^.

Moreover, the inverse problem of fracture estimation is ill-conditioned, and the inversion results will be unstable without any constraint to the problem. The estimation of sensitive fracture weaknesses should be implemented under the constraints of regularization terms^15^. Therefore, we attempt to use the PP-wave seismic reflected amplitude data to estimate the normal and shear weaknesses ($\delta_{N}$ and $\delta_{T}$) of fractures with the regularization constraints in joint time–frequency domain. Integrating the relationship between Thomsen-type anisotropic parameters and fracture weakness parameters^26^, we first construct the forward modelling equation following Rüger’s weak-anisotropy PP-wave reflection coefficient^12,13^. Then a Bayesian framework is introduced to the inversion for sensitive fracture weaknesses in joint time–frequency domain, which combines the prior constraint information and sparse-distribution likelihood function to estimate the posterior distribution of fracture parameters. In this paper, we construct the cost function using the assumption of a Cauchy-distribution prior constraint and a Gaussian-distribution likelihood function^15,27^. In addition, a low-frequency smoothing model constraint is also introduced to the cost function to obtain more stable estimation of Bayesian AVOA inversion^15,28^. We finally present a method of azimuth-dependent and azimuthal-seismic-amplitude-difference-based inversion to estimate the fracture parameters in joint time–frequency domain. The iteratively reweighted least-squares (IRLS) algorithm to solve the inversion problem for fracture estimation^29,30^. Synthetic data examples demonstrate that the normal and shear fracture weaknesses can be reasonably and reliably inverted when the PP-wave azimuthal seismic data contains moderate or even relatively high random noises. The real data set acquired over a fractured reservoir further validate that our proposed inversion approach in joint time–frequency domain can achieve the fracture detection from the azimuth-dependent PP-wave seismic data.

## Methods

### Forward matrix in time–frequency domain

Following Rüger’s weak-anisotropy equation for an horizontal transversely isotropic (HTI) medium and the relationship between Thomsen’s anisotropic parameters and fracture weaknesses^26^, the azimuth-related linearized PP-wave reflection coefficient $R_{PP}^{HTI} \left( {\theta ,\varphi } \right)$ for an interface separating two HTI media can be written as in the form^15^,

1 $$ R_{PP}^{HTI} \left( {\theta ,\varphi } \right){ = }R_{PP}^{ISO} \left( \theta \right) + R_{PP}^{ANI} \left( {\theta ,\varphi } \right), $$

where $\theta$ and $\varphi$ are the angles of incidence and azimuth, respectively; $R_{PP}^{ISO} \left( \theta \right)$ is the azimuth-independent background isotropic reflection coefficient, and $R_{PP}^{ANI} \left( {\theta ,\varphi } \right)$ is the fracture-induced and azimuth-dependent reflection coefficient in an HTI medium formed by a single set of vertical and rotationally invariant fractures embedded in a homogeneously isotropic background rocks, which is given by

2 $$ R_{PP}^{ANI} \left( {\theta ,\varphi } \right){ = }a_{{\delta_{N} }} \left( {\theta ,\varphi } \right)\Delta \delta_{N} + a_{{\delta_{T} }} \left( {\theta ,\varphi } \right)\Delta \delta_{T} , $$

where the symbol $\Delta$ represents the value changes of normal and shear fracture weaknesses ($\delta_{N}$ and $\delta_{T}$) between upper and lower layers separated by the reflection interface, and the weighting coefficients of fracture weaknesses ($a_{{\delta_{N} }}$ and $a_{{\delta_{T} }}$) can be expressed as

3 $$ a_{{\delta_{N} }} \left( {\theta ,\varphi } \right){ = } - g\cos^{2} \varphi \sin^{2} \theta \left[ {\left( {1 - 2g} \right)\left( {1 + \sin^{2} \varphi \tan^{2} \theta } \right) + \left( {1 - g} \right)\left( {\cos^{2} \varphi \tan^{2} \theta } \right)} \right], $$

and

4 $$ a_{{\delta_{T} }} \left( {\theta ,\varphi } \right){ = }g\cos^{2} \varphi \sin^{2} \theta \left( {1 - \sin^{2} \varphi \tan^{2} \theta } \right). $$

Here $g$ represents the square of S-to-P-wave velocity ratio of media. In the following paper, $g$ used in the synthetic data examples is the ratio of the square of well log S-wave velocity and P-wave velocity, and $g$ used in the field data example is the ratio of the square of initial S-wave velocity and P-wave velocity model.

To obtain the azimuth-dependent anisotropic parameters, we can just utilize the fracture-induced and azimuth-dependent reflection coefficient $R_{PP}^{ANI} \left( {\theta ,\varphi } \right)$ to estimate the fracture weaknesses, which can be used for fracture detection. Integrating the estimated seismic wavelets, the vector of azimuth-dependent seismic reflection data in time domain (for example, two azimuths, three incidence angles, and $M$ reflected interfaces) can be written as in the form,

5 $$ {\mathbf{d}}_{t} = {\mathbf{WR}}_{PP}^{ANI} = {\mathbf{WAm}} = {\mathbf{G}}_{t} {\mathbf{m}}, $$

where ${\mathbf{d}}_{t}$ is the azimuth-dependent seismic difference data vector, ${\mathbf{A}}$ is the weight coefficient matrix of model parameters, ${\mathbf{m}}$ is the target model matrix, and ${\mathbf{G}}_{t} = {\mathbf{WA}}$ is the product of the seismic wavelet matrix and the weight coefficient matrix of model parameters, which can be expressed as

6 $$ \left[ {{\mathbf{d}}_{t} } \right]_{3M \times 1} = \left[ {\begin{array}{*{20}c} {d\left( {t_{1} ,\theta_{i} ,\varphi_{2} } \right) - d\left( {t_{1} ,\theta_{i} ,\varphi_{1} } \right)} & {\cdots} & {d\left( {t_{M} ,\theta_{i} ,\varphi_{2} } \right) - d\left( {t_{M} ,\theta_{i} ,\varphi_{1} } \right)} \\ \end{array} } \right]^{T} ,\left( {i = 1,2,3} \right) $$

7 $$ {\mathbf{A}}_{3M \times 2M} = \left[ {\begin{array}{*{20}c} {diag\left[ {\begin{array}{*{20}c} {a_{{\delta_{N} }}^{{}} \left( {t_{1} ,\theta_{i} ,\varphi_{2} } \right) - a_{{\delta_{N} }}^{{}} \left( {t_{1} ,\theta_{i} ,\varphi_{1} } \right)} & {...} & {a_{{\delta_{N} }}^{{}} \left( {t_{M} ,\theta_{i} ,\varphi_{2} } \right) - a_{{\delta_{N} }}^{{}} \left( {t_{M} ,\theta_{i} ,\varphi_{1} } \right)} \\ \end{array} } \right]^{T} } \\ {diag\left[ {\begin{array}{*{20}c} {a_{{\delta_{T} }}^{{}} \left( {t_{1} ,\theta_{i} ,\varphi_{2} } \right) - a_{{\delta_{T} }}^{{}} \left( {t_{1} ,\theta_{i} ,\varphi_{1} } \right)} & {\cdots} & {a_{{\delta_{T} }}^{{}} \left( {t_{M} ,\theta_{i} ,\varphi_{2} } \right) - a_{{\delta_{T} }}^{{}} \left( {t_{M} ,\theta_{i} ,\varphi_{1} } \right)} \\ \end{array} } \right]^{T} } \\ \end{array} } \right]^{T} ,\left( {i = 1,2,3} \right) $$

8 $$ {\mathbf{m}}_{2M \times 1} = \left[ {\begin{array}{*{20}c} {\left[ {\begin{array}{*{20}c} {\Delta \delta_{N} \left( {t_{1} } \right)} & {...} & {\Delta \delta_{N} \left( {t_{M} } \right)} \\ \end{array} } \right]^{T} } \\ {\left[ {\begin{array}{*{20}c} {\Delta \delta_{T} \left( {t_{1} } \right)} & {...} & {\Delta \delta_{T} \left( {t_{M} } \right)} \\ \end{array} } \right]^{T} } \\ \end{array} } \right], $$

and $${\mathbf{W}} = \left[ {\begin{array}{*{20}c} {w_{1} } & 0 & 0 & {...} \\ {w_{2} } & {w_{1} } & 0 & \ddots \\ {w_{3} } & {w_{2} } & {w_{1} } & \ddots \\ \vdots & \ddots & \ddots & \ddots \\ \end{array} } \right]$$ is the wavelet matrix, and $w_{j}$ denotes the *j*th term of an extracted seismic wavelet; $${\mathbf{R}}_{PP}^{ANI} = \left[ {\begin{array}{*{20}c} {R_{PP}^{ANI} \left( {t_{1} ,\theta_{i} ,\varphi_{2} } \right) - R_{PP}^{ANI} \left( {t_{1} ,\theta_{i} ,\varphi_{1} } \right)} & {...} & {R_{PP}^{ANI} \left( {t_{M} ,\theta_{i} ,\varphi_{2} } \right) - R_{PP}^{ANI} \left( {t_{M} ,\theta_{i} ,\varphi_{1} } \right)} \\ \end{array} } \right]^{T}$$ is the matrix of reflection coefficient, respectively, and the symbol $T$ represents the transposition of a matrix. In contrast, the seismic data ${\mathbf{d}}\left( \omega \right)$ in frequency domain can be written as

9 $$ {\mathbf{d}}\left( \omega \right) = {\mathbf{W}}\left( \omega \right){\mathbf{R}}\left( \omega \right), $$

where $\omega$ is the angular frequency, ${\mathbf{W}}\left( \omega \right)$ is the frequency spectrum of seismic wavelets, and ${\mathbf{R}}\left( \omega \right)$ is the frequency spectrum of the fracture-induced and azimuth-dependent reflection coefficient $R_{PP}^{ANI} \left( {\theta ,\varphi } \right)$, which can be expressed as

10 $$ {\mathbf{R}}\left( \omega \right){ = }\int\limits_{0}^{{{ + }\infty }} {R_{PP}^{ANI} \left( z \right)\exp \left[ { - j\omega \tau \left( z \right)} \right]dz} , $$

where $\tau \left( z \right)$ denotes the time-domain depth, and $\exp \left( \cdot \right)$ is an exponential function. Equation (9) can be rewritten as in the form,

11 $$ {\mathbf{d}}_{f} = {\mathbf{G}}_{f} {\mathbf{m}}{ = }{\mathbf{W}}\left( \omega \right){\mathbf{AE}}\left( \omega \right){\mathbf{m}}, $$

where ${\mathbf{E}}\left( \omega \right)$ represents the Fourier transform operator or the time shift operator.

### Bayesian inference in time–frequency domain

Bayesian inference in seismic inversion can be used to establish the a posteriori probability density function (PDF) as a product of the a priori PDF and the likelihood function^27^. The likelihood function depends on the PDF of background seismic noises. Assuming that the seismic data in time domain and data in frequency domain are both independent random variables^23,27^, the joint likelihood function in time–frequency domain can be expressed as

12 $$ p\left( {\left. {{\mathbf{d}}_{t} ,{\mathbf{d}}_{f} } \right|{\mathbf{m}}} \right) = p\left( {\left. {{\mathbf{d}}_{t} } \right|{\mathbf{m}}} \right) \cdot p\left( {\left. {{\mathbf{d}}_{f} } \right|{\mathbf{m}}} \right), $$

where $p\left( \cdot \right)$ represents a PDF, and the three PDFs denote the degree of matching between inversion results and seismic data in joint time–frequency domain, time domain, and frequency domain, respectively. We further assume that the likelihood functions of seismic data ${\mathbf{d}}_{t}$ and ${\mathbf{d}}_{f}$ in time domain and frequency domain both satisfy the Gaussian PDF with mean zero, and the joint likelihood function can be expressed as

13 $$ p_{Gauss} \left( {\left. {{\mathbf{d}}_{t} ,{\mathbf{d}}_{f} } \right|{\mathbf{m}}} \right) = \left\{ {\frac{1}{{\left( {2\pi \sigma_{t}^{2} } \right)^{{{M \mathord{\left/ {\vphantom {M 2}} \right. \kern-\nulldelimiterspace} 2}}} }} \cdot \exp \left[ {\frac{{ - \left\| {{\mathbf{d}}_{t} - {\mathbf{G}}_{t} {\mathbf{m}}} \right\|_{2}^{2} }}{{2\sigma_{t}^{2} }}} \right]} \right\} \cdot \left\{ {\frac{1}{{\left( {2\pi \sigma_{f}^{2} } \right)^{{{K \mathord{\left/ {\vphantom {K 2}} \right. \kern-\nulldelimiterspace} 2}}} }} \cdot \exp \left[ {\frac{{ - \left\| {{\mathbf{d}}_{f} - {\mathbf{G}}_{f} {\mathbf{m}}} \right\|_{2}^{2} }}{{2\sigma_{f}^{2} }}} \right]} \right\}, $$

where the symbol $\left\| \cdot \right\|_{2}$ represents 2-norm function, $\sigma_{t}^{2}$ and $\sigma_{f}^{2}$ are the variances of time-domain and frequency-domain seismic data, respectively. Equation (13) links the seismic response between time-domain and frequency-domain data. The a priori PDF of unknown model parameters is used to describe the prior information of model parameters, and Cauchy distribution is utilized as the a *priori* PDF, which is given by

14 $$ p_{Cauchy} \left( {\mathbf{m}} \right) = \frac{1}{{\left( {\pi \sigma_{{\mathbf{m}}}^{2} } \right)^{M} }}\prod\limits_{i = 1}^{M} {\frac{1}{{1{ + }{{{\mathbf{m}}_{i}^{2} } \mathord{\left/ {\vphantom {{{\mathbf{m}}_{i}^{2} } {\sigma_{{\mathbf{m}}}^{2} }}} \right. \kern-\nulldelimiterspace} {\sigma_{{\mathbf{m}}}^{2} }}}}} , $$

where $\sigma_{{\mathbf{m}}}^{2}$ is the variance of model parameter. Based on the Bayesian inference, the joint *a* posteriori PDF $p\left( {\left. {\mathbf{m}} \right|{\mathbf{d}}_{t} ,{\mathbf{d}}_{f} } \right)$ can be given by^28^

15 $$ p\left( {\left. {\mathbf{m}} \right|{\mathbf{d}}_{t} ,{\mathbf{d}}_{f} } \right){ = }\frac{{p_{Cauchy} \left( {\mathbf{m}} \right)p_{Gauss} \left( {\left. {{\mathbf{d}}_{t} ,{\mathbf{d}}_{f} } \right|{\mathbf{m}}} \right)}}{{\int {p_{Cauchy} \left( {\mathbf{m}} \right)p_{Gauss} \left( {\left. {{\mathbf{d}}_{t} ,{\mathbf{d}}_{f} } \right|{\mathbf{m}}} \right)d{\mathbf{m}}} }} \propto p_{Cauchy} \left( {\mathbf{m}} \right)p_{Gauss} \left( {\left. {{\mathbf{d}}_{t} ,{\mathbf{d}}_{f} } \right|{\mathbf{m}}} \right), $$

that is,

16 $$ p\left( {\left. {\mathbf{m}} \right|{\mathbf{d}}_{t} ,{\mathbf{d}}_{f} } \right) \propto \prod\limits_{i = 1}^{M} {\frac{1}{{1{ + }{{{\mathbf{m}}_{i}^{2} } \mathord{\left/ {\vphantom {{{\mathbf{m}}_{i}^{2} } {\sigma_{{\mathbf{m}}}^{2} }}} \right. \kern-\nulldelimiterspace} {\sigma_{{\mathbf{m}}}^{2} }}}}} \cdot \exp \left[ { - \frac{{\left\| {{\mathbf{d}}_{t} - {\mathbf{G}}_{t} {\mathbf{m}}} \right\|_{2}^{2} }}{{2\sigma_{t}^{2} }} - \frac{{\left\| {{\mathbf{d}}_{f} - {\mathbf{G}}_{f} {\mathbf{m}}} \right\|_{2}^{2} }}{{2\sigma_{f}^{2} }}} \right]. $$

Maximizing the joint a *posteriori* PDF $p\left( {\left. {\mathbf{m}} \right|{\mathbf{d}}_{t} ,{\mathbf{d}}_{f} } \right)$ in Eq. (16), we obtain the objective function $J\left( {\mathbf{m}} \right)$, which is given by

17 $$ J\left( {\mathbf{m}} \right){ = }J_{Gauss} \left( {\mathbf{m}} \right){ + }J_{Cauchy} \left( {\mathbf{m}} \right){ = }\left\| {{\mathbf{d}}_{t} - {\mathbf{G}}_{t} {\mathbf{m}}} \right\|_{2}^{2} { + }\chi_{{1}} \left\| {{\mathbf{d}}_{f} - {\mathbf{G}}_{f} {\mathbf{m}}} \right\|_{2}^{2} { + }\chi_{{2}} \sum\limits_{i = 1}^{M} {\ln \left( {1{ + }{{{\mathbf{m}}_{i}^{2} } \mathord{\left/ {\vphantom {{{\mathbf{m}}_{i}^{2} } {\sigma_{{\mathbf{m}}}^{2} }}} \right. \kern-\nulldelimiterspace} {\sigma_{{\mathbf{m}}}^{2} }}} \right)} , $$

where $J_{Gauss} \left( {\mathbf{m}} \right)$ denotes the measurement of the difference between seismic response in joint time–frequency domain and forwarding synthesized gathers, and $J_{Cauchy} \left( {\mathbf{m}} \right)$ denotes the sparse constraint regularization term introduced by the *a* priori PDF term; $\chi_{{1}} { = }{{\sigma_{t}^{2} } \mathord{\left/ {\vphantom {{\sigma_{t}^{2} } {\sigma_{f}^{2} }}} \right. \kern-\nulldelimiterspace} {\sigma_{f}^{2} }}$ and $\chi_{{2}} { = 2}\sigma_{t}^{2}$ are the regularization coefficients of seismic data error in frequency domain and sparse constraint term, respectively. Moreover, we introduce the low-frequency-model constraint regularization term $J_{\bmod } \left( {\mathbf{m}} \right)$ into the objective function in Eq. (17) which can be written as

18 $$ \begin{gathered} J_{ALL} \left( {\mathbf{m}} \right){ = }J_{Gauss} \left( {\mathbf{m}} \right){ + }J_{Cauchy} \left( {\mathbf{m}} \right){ + }J_{\bmod } \left( {\mathbf{m}} \right) \hfill \\ { = }\left\| {{\mathbf{d}}_{t} - {\mathbf{G}}_{t} {\mathbf{m}}} \right\|_{2}^{2} { + }\chi_{{1}} \left\| {{\mathbf{d}}_{f} - {\mathbf{G}}_{f} {\mathbf{m}}} \right\|_{2}^{2} { + }\chi_{{2}} \sum\limits_{i = 1}^{M} {\ln \left( {1{ + }{{{\mathbf{m}}_{i}^{2} } \mathord{\left/ {\vphantom {{{\mathbf{m}}_{i}^{2} } {\sigma_{{\mathbf{m}}}^{2} }}} \right. \kern-\nulldelimiterspace} {\sigma_{{\mathbf{m}}}^{2} }}} \right)} { + }\chi_{{3}} \sum\limits_{i = 1}^{M} {\left\| {{{\varvec{\upzeta}}} - {\mathbf{Pm}}_{i} } \right\|_{2}^{2} } , \hfill \\ \end{gathered} $$

where $\chi_{{3}}$ denotes regularization coefficient of low-frequency-model constraint, ${{\varvec{\upzeta}}}$ and ${\mathbf{P}}$ are the low-frequency smoothing models of unknown model parameters and the integral matrix, respectively. Minimizing the final objective function $J_{ALL} \left( {\mathbf{m}} \right)$, we can get the nonlinear inversion equation. Here we use the iteratively reweighted least-squares (IRLS) algorithm to solve the nonlinear Eq. (18) iteratively^29,30^. After a couple of iterations, the IRLS algorithm can reach the state of convergence. When using the IRLS algorithm, some steps are demanded to implement the nonlinear and iterative inversion, including the construction of initial model parameters, the selection of iteration times, and the setting of convergence threshold. We then calculate the objective function iteratively, and finally obtain the inversion results according to the iteration times or the convergence threshold.

## Results and discussions

**Figure 1:**
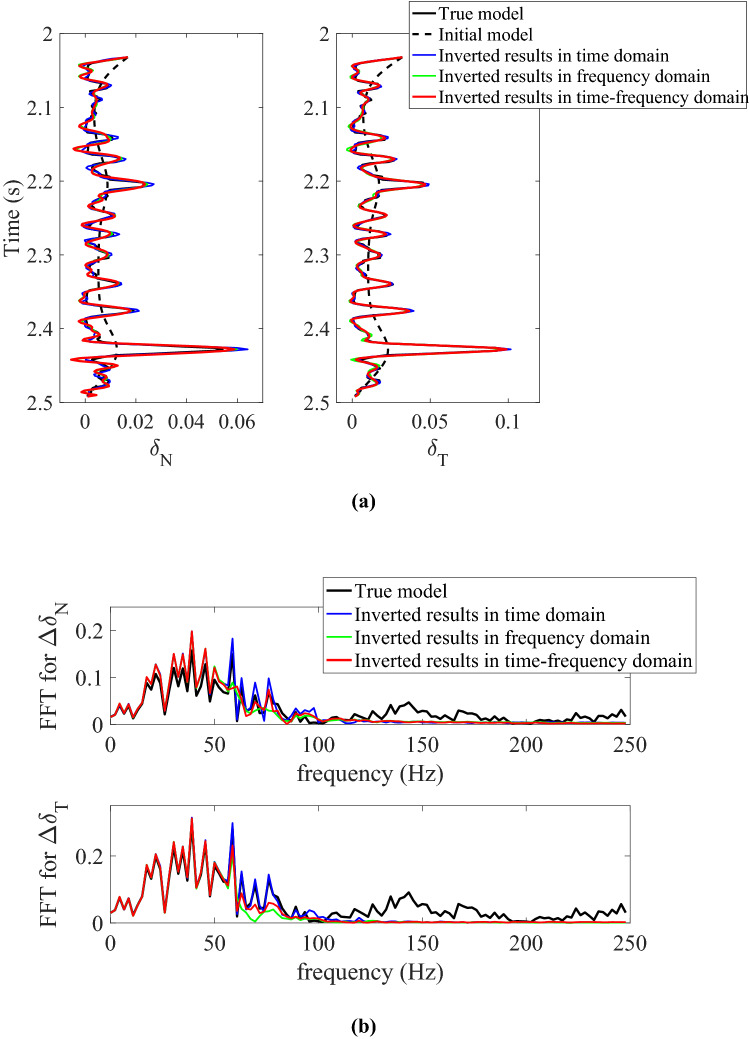
Comparison between original and inverted model parameters and spectra in time domain, frequency domain, and joint time–frequency domain with synthetic azimuth-dependent seismic data containing no noises, where **(a)** is the inverted normal and shear fracture weaknesses in different domains, and **(b)** is the spectra comparison in different domains.

To validate the proposed approach, we first use the synthetic data generated by PP-wave reflection coefficient (computed with Eq. 1) convoluted with seismic wavelets without noises, and then perform the azimuth-dependent and azimuthal-amplitude-difference-based seismic inversion for normal and shear weaknesses. Figure 1a shows the inversion results in time domain (blue curves), frequency domain (green curves), and joint time–frequency domain (red curves), respectively, and the initial model (dotted black curves) are generated by smoothing the true well log data (solid black curves). We find that the inverted fracture weaknesses are all consistent with the true values in all three domains. Figure 1b shows the corresponding spectra of differences in normal (above) and shear (below) fracture weaknesses, respectively, and the spectra of inverted results in all three domains are still a good match in the seismic band. Therefore, the inversion methods in all three domains perform well when seismic data contains no noises.

**Figure 2:**
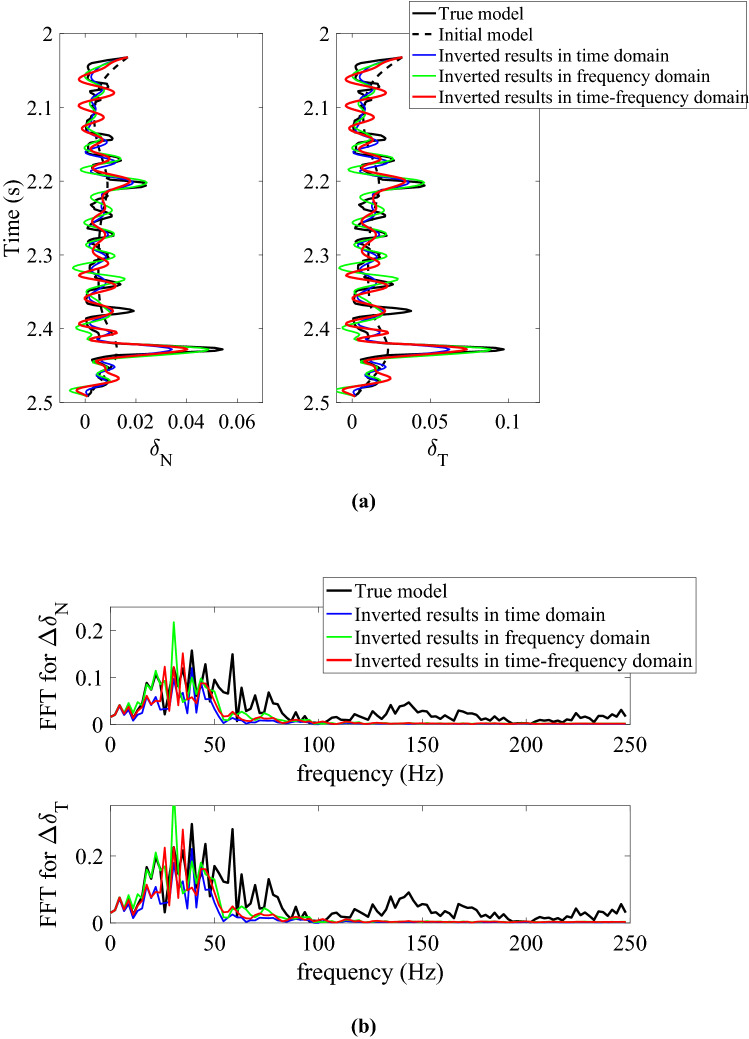
Comparison between original and inverted model parameters and spectra in time domain, frequency domain, and joint time–frequency domain with synthetic azimuth-dependent seismic data containing moderate noises (SNR = 5), where **(a)** is the inverted normal and shear fracture weaknesses in different domains, and **(b)** is the spectra comparison in different domains.

**Figure 3:**
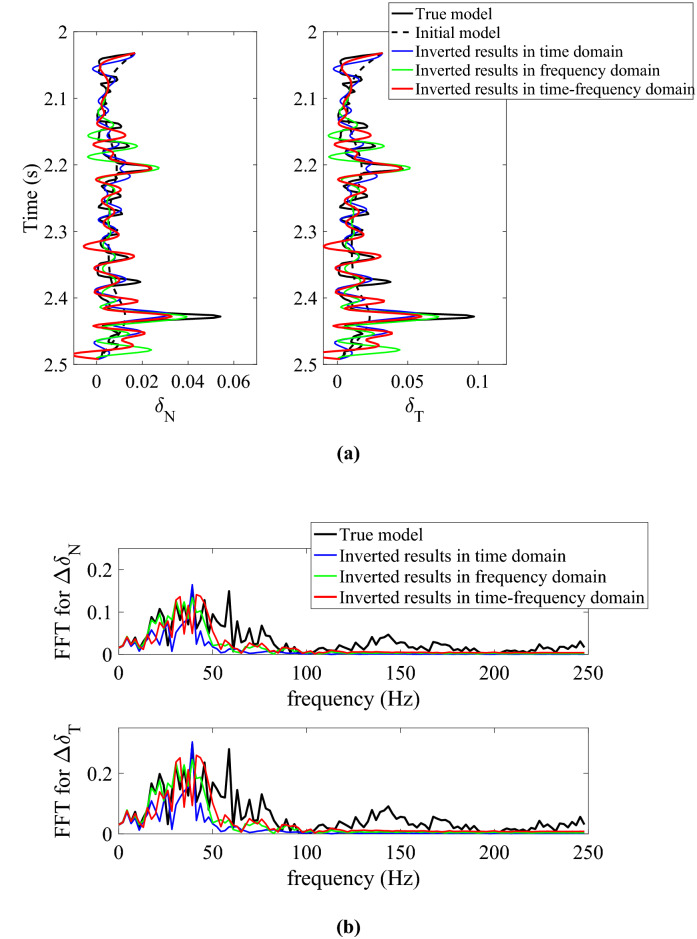
Comparison between original and inverted model parameters and spectra in time domain, frequency domain, and joint time–frequency domain with synthetic azimuth-dependent seismic data containing more noises (SNR = 2), where **(a)** is the inverted normal and shear fracture weaknesses in different domains, and **(b)** is the spectra comparison in different domains.

To further test the anti-noise ability of inversion methods in different domains, we add moderate Gaussian random white noises into the noise-free data and generate the synthetic data with different signal-to-noise-ratios (SNRs) being 5 and 2, respectively. We then perform the azimuth-dependent and azimuthal-amplitude-difference-based seismic inversion for normal and shear weaknesses in noisy cases. Figure 2a,b show the comparison between original and inverted model parameters and spectra in time domain (blue lines), frequency domain (green lines), and joint time–frequency domain (red lines) with synthetic azimuth-dependent seismic data containing moderate noises (i.e., the SNR of data is 5). From the inversion results in different domains shown in Fig. 2a, we can see that the inversion accuracy of fracture weaknesses in frequency and joint time–frequency domain are better that in time domain, and the inversion results in joint time–frequency domain are more stable than that in frequency domain from the spectra comparison shown in Fig. 2b. Figure 3a,b show the same case but with more noises (i.e., the SNR of data is 2), and we can find that the spectra of inverted fracture weaknesses in joint time–frequency domain are wider than the other inversion results in time or frequency domains. To validate the stability of the proposed inversion approach, we compare the cross-correlation coefficients between the true and inverted results in time, frequency and joint time–frequency domains. Table 1 illustrates the comparison results, and we can obviously find that the cross-correlation coefficients between true and inverted fracture weaknesses in joint time–frequency domain are larger than that in time and frequency domains when the seismic data contains moderate or even more noises. Therefore, the inversion method in joint time–frequency domain maintains a balance between the anti-noise ability and resolution effect.

**Table 1:** Cross-correlation coefficients between true and inverted fracture weaknesses in time, frequency, and joint time–frequency domain.

Cross-correlation coefficients		Noise-free case	SNR = 5 case	SNR = 2 case
Time-domain inversion	$\delta_{N}$	0.9712	0.8795	0.7655
	$\delta_{T}$	0.9841	0.8750	0.7637
Frequency-domain inversion	$\delta_{N}$	0.9602	0.8334	0.7558
	$\delta_{T}$	0.9921	0.8266	0.7495
Joint time–frequency domain inversion	$\delta_{N}$	0.9897	0.8927	0.7868
	$\delta_{T}$	0.9956	0.8882	0.7783

**Figure 4:**
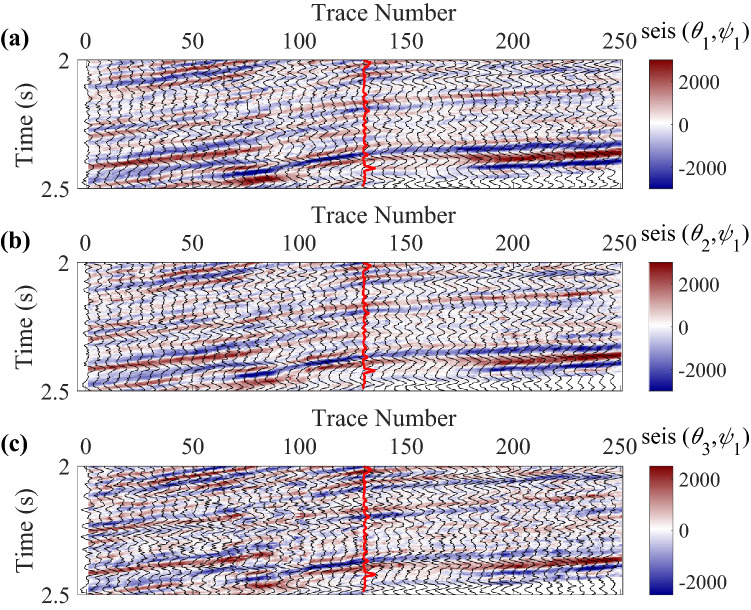
Angle-stacked seismic data in azimuth $\varphi_{1} { = }40^{\circ}$, where **(a)** is the near angle of incidence (an average of 18° ranging from 14° to 22°), **(b)** is the middle angle of incidence (an average of 22° ranging from 18° to 26°), and **(c)** is the far angle of incidence (an average of 26° ranging from 22° to 30°), respectively.

**Figure 5:**
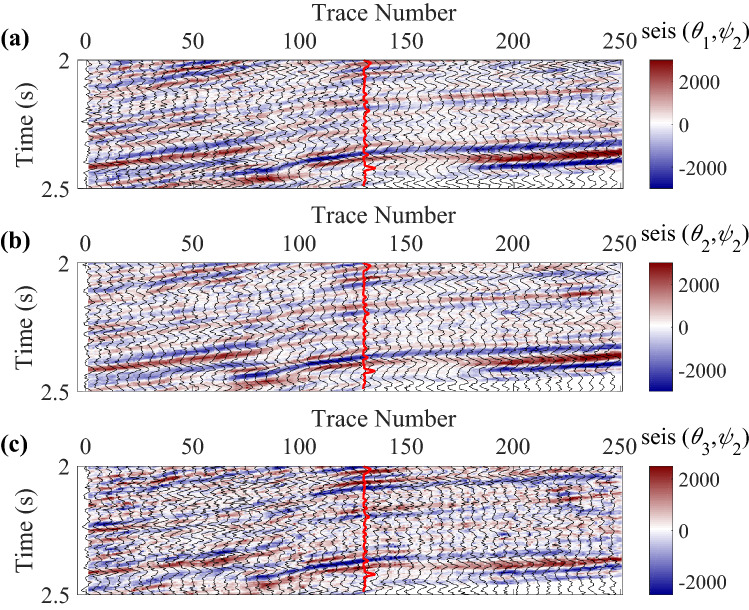
Angle-stacked seismic data in the other azimuth $\varphi_{2} { = }130^{\circ}$, where **(a)** is the near angle of incidence, **(b)** is the middle angle of incidence, and **(c)** is the far angle of incidence, respectively.

**Figure 6:**
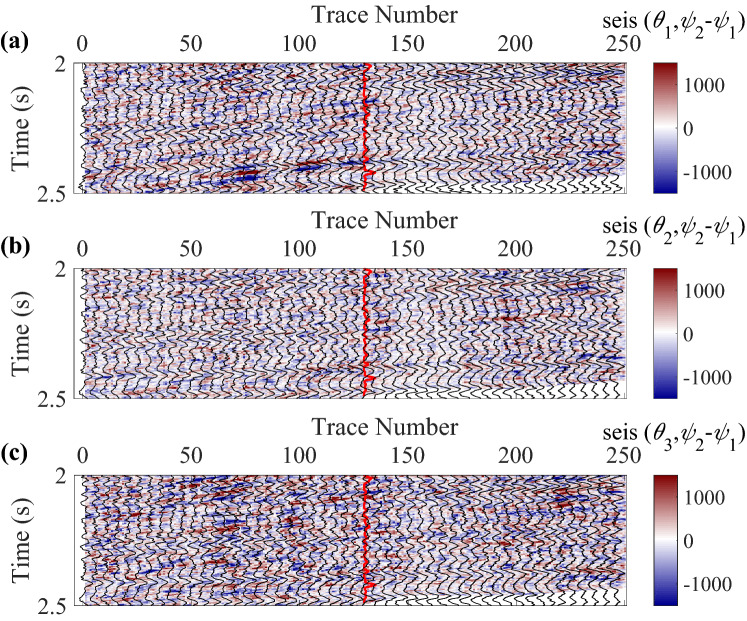
Seismic amplitude difference data between azimuth $\varphi_{1} { = }40^{\circ}$ and azimuth $\varphi_{2} { = }130^{\circ}$, where **(a)** is the near angle of incidence, **(b) **is the middle angle of incidence, and **(c)** is the far angle of incidence, respectively.

A real data acquired from a fractured reservoir in Sichuan Basin, China is also used to further demonstrate the proposed inversion method, which is a wide-azimuth land survey dataset. To quickly estimate the normal and shear fracture weaknesses, we use two azimuth-dependent seismic data with three angles of incidence to implement the azimuth-dependent and azimuthal-amplitude-difference-based seismic inversion for fracture detection in joint time–frequency domain. The azimuth of fracture normal was first calculated using the least-squares ellipse fitting method, and then the two azimuths were selected based on the estimated fracture normal to obtain the large seismic amplitude differences. Of course, the method can easily be extended to the multi azimuth data easily just changing the azimuth-dependent seismic difference data vector, the weight coefficient matrix of model parameters, and the wavelet matrix. However, the inversion with two azimuths is simple and generally gives acceptable results in practice, and we attempt to implement the fracture detection using only two azimuth data to simplify the inversion processing. The trace spacing is 20 m. Before the seismic inversion, the data are processed to guarantee that the finely processed data is high-quality enough to be used for amplitude versus offset and azimuth (AVOA) inversion. The workflow of data processing is presented in Table 2, and details about the data processing are presented in Dulaijian^32^. Figures 4 and 5 are the azimuth-dependent seismic data with near, middle, and far angles of incidence generated from angle-stacked seismic data. The main frequencies of data vary from 10 to 50 Hz in this work area, and we use three inversion methods to estimate the fracture weaknesses in time, frequency, and joint time–frequency domains, respectively. All three methods are based on the information of seismic amplitude difference in different azimuth, and Fig. 6 shows the corresponding amplitude difference data. Note that the red curves in data profiles are the well log curves of shear weakness, which can be represented as the fracture density. Next we perform the proposed inversion method to characterize the fractured reservoirs.

**Table 2:** Workflow of data processing for the azimuthal PP-wave seismic data.

1. Azimuthal PP-wave data reading and editing
2. Format transformation of dataset
3. Trace editing and regularization of dataset 4. Static correction of dataset 5. Significant noise suppressed of dataset in multiple domains 6. Spherical divergence correlation of dataset 7. Surface-consistent amplitude correction of dataset 8. Deconvolution of dataset 9. Muting of dataset 10. Sort common mid point (CMP) of data traces 11. Velocity analysis of dataset
12. Normal moveout (NMO) of dataset
13. Dip moveout (DMO) of dataset 14. Residual static correlation for common-azimuth varying-offset gathers 15. Pre-stack time migration of dataset 16. Inverse NMO of dataset 17. New velocity picking of dataset 18. NMO with new velocity 19. New residual static correlation and surface-consistent amplitude processing
20. Sectoring pre-stack data into azimuthal sectors, or by COV binning 21. Isotropic and anisotropic migration velocity analysis 22. Trim static correlation
23. Transform to time domain 24. Stack to generate three partial angle-stack seismic volumes with multiple azimuths

**Figure 7:**
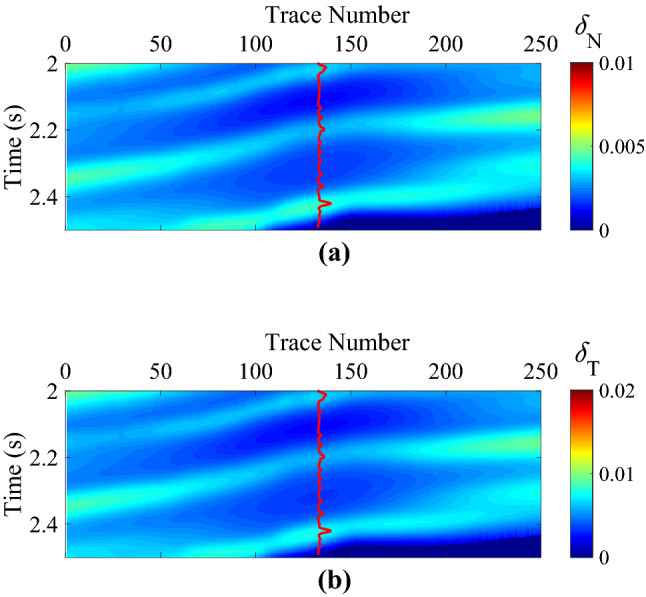
Initial models of normal and shear fracture weaknesses, where **(a)** is the normal fracture weakness, and **(b)** is the shear fracture weakness, respectively.

**Figure 8:**
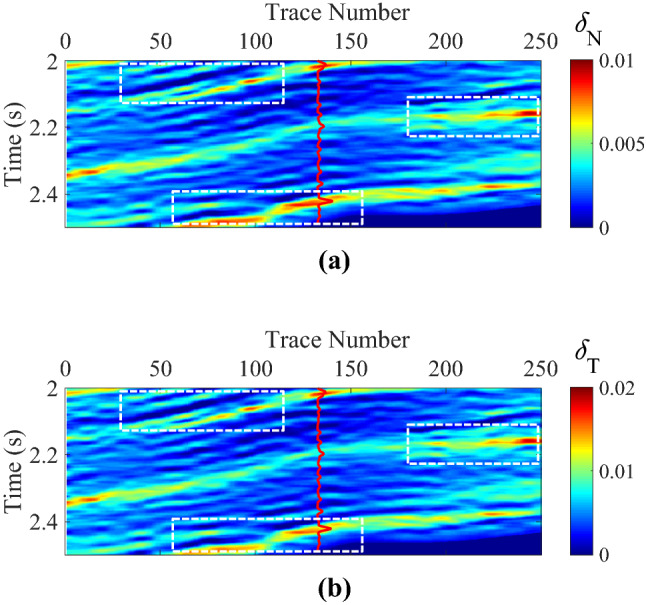
Inverted normal and shear fracture weaknesses in time domain, where **(a)** is the normal fracture weakness, and **(b)** is the shear fracture weakness, respectively.

**Figure 9:**
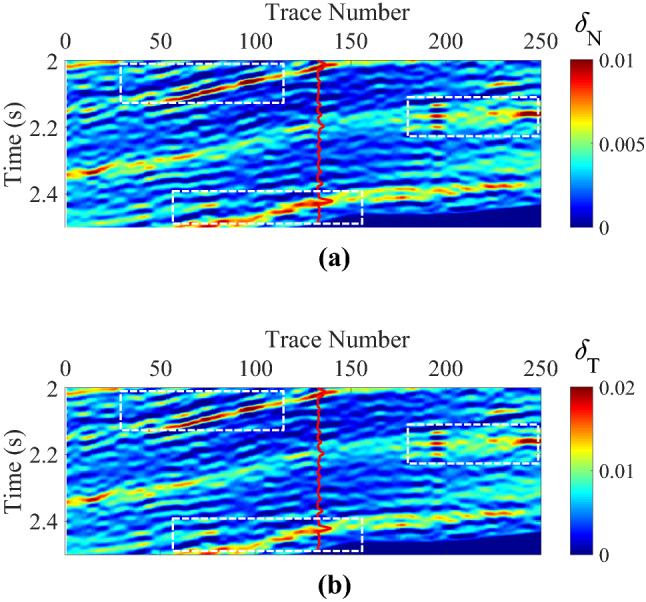
Inverted normal and shear fracture weaknesses in frequency domain, where **(a)** is the normal fracture weakness, and **(b)** is the shear fracture weakness, respectively.

**Figure 10:**
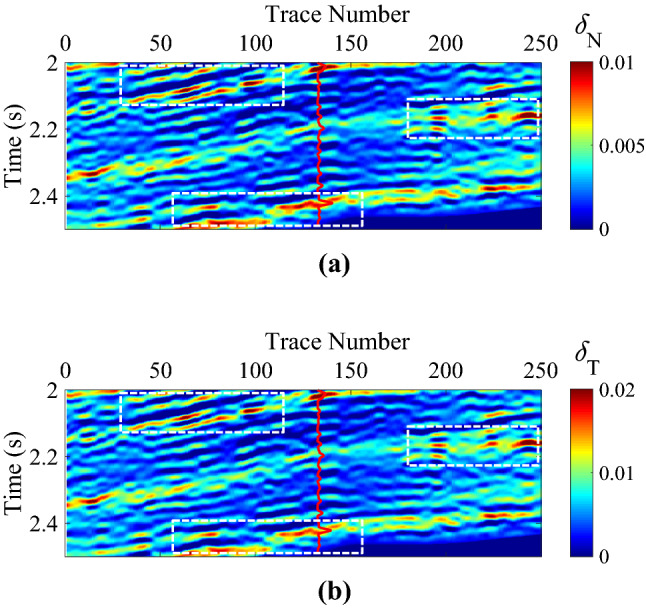
Inverted normal and shear fracture weaknesses in joint time–frequency domain, where **(a)** is the normal fracture weakness, and **(b)** is the shear fracture weakness, respectively.

The interpreted well-log fracture density information in this work area was not available. We estimated the fracture weaknesses based on an azimuthally anisotropic rock-physics model with conventional well log data^15^. Figure 7a,b are the initial models of normal and shear fracture weaknesses, and the curves are the corresponding estimated information of fracture weaknesses. Of course, the estimated fracture weakness parameters should be calibrated by using the interpreted well-log fracture density information and the image logging, especially the micro-resistivity image logging (FMI). In this work area, there is no anisotropic well log information available, such as the interpreted well-log fracture density information, but the FMI information can be interpreted as the initial constraint of the estimation of the fracture development situation. Figure 8a,b are the inverted fracture weaknesses in time domain, Fig. 9a,b are the inverted fracture weaknesses in frequency domain, and Fig. 10a,b are the inverted fracture weaknesses in joint time–frequency domain, respectively.

**Figure 11:**
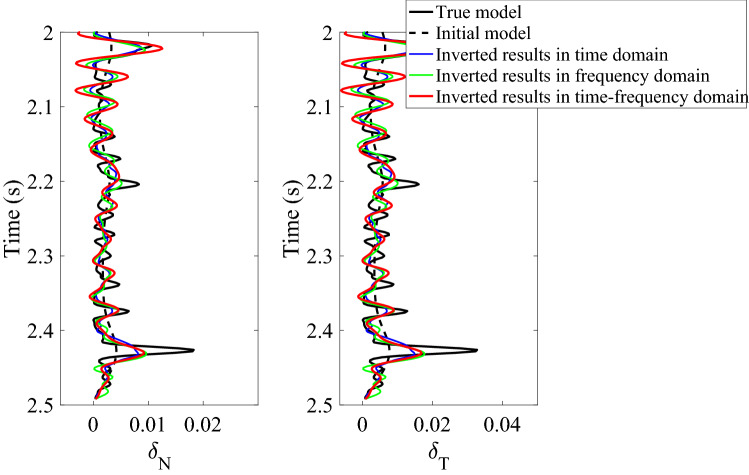
Comparison between inversion results of normal and shear fracture weaknesses in different domain at the well location.

**Figure 12:**
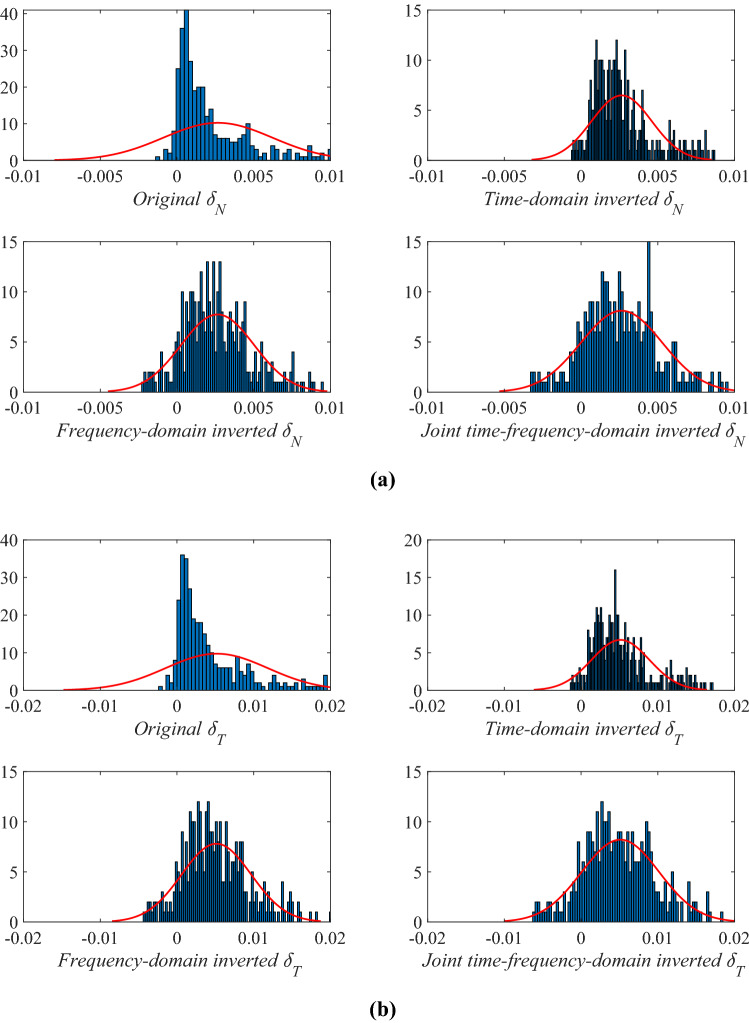
Comparison of histograms between original and inverted normal and shear weaknesses in time, frequency, and joint time–frequency domains, where **(a)** shows the results of normal weakness, and **(b)** shows the results of shear weakness.

High-value fracture weaknesses illustrate the developed fractures in reservoirs. From the above inversion results of fracture weaknesses in different domains, we find that the inversion results in frequency domain or in joint-time–frequency domain show higher resolution compared with the inverted results in time domain, but the time-domain inversion results exhibit better lateral continuities. However, the joint-time–frequency-domain inversion results may provide more geologically reasonable interpretations for the fractured reservoirs in this area due to the discontinuous reservoirs of fracture development. Figure 11 is the comparison between inversion results in different domain at the well location, and we also find that the time–frequency-domain inversion method can achieve a balance between the anti-noise ability and seismic resolution. Figure 12 illustrates the comparison of histograms between original and inverted normal and shear weaknesses in time, frequency, and joint time–frequency domains, and we find that the a *posteriori* PDF of inverted fracture weaknesses in all three domains nearly agrees the Gaussian distribution with the a priori PDF as we have assumed.

## Conclusions

Motivated by fracture detection in fractured reservoirs based on azimuth-dependent seismic inversion, we establish an inversion method by integrating Bayesian inference and regularization constraints in joint time–frequency domain to estimate the normal and shear fracture weaknesses. Combining the azimuth-dependent seismic amplitude difference information, we express the a posteriori probability distribution as a product of the a priori probability distribution and the likelihood function, and get the objective function by maximizing the a posteriori probability distribution. We finally estimate the characteristic parameters of fracture properties iteratively via a reweighted least-squares algorithm. Compared with the time- and frequency-domain inversion results, the joint time–frequency-domain inversion method gets more accurate and high-resolution results. It shows that the joint time–frequency-domain inversion method achieve a balance between the anti-noise ability and resolution effect compared with the time-domain and frequency-domain inversion. The cross-correlation coefficients between true model and inverted results in time, frequency, and joint time–frequency domains further validates the conclusion quantificationally. The test on a field data demonstrates that the proposed approach can obtain more accurate and robust inversion results than that in a separate time or frequency domain, in which the high-value fracture weaknesses are used to characterize the development areas of fractures. Therefore, the proposed inversion approach may provide a new way to perform the fracture detection by combining the different domain information for the seismic data.

## Data Availability

The datasets used during the current study are available from the corresponding author on reasonable request.

## Appendix A

Following the assumption that a porous fractured medium can be taken as a periodic horizontally stratified layer, the normal and shear fracture compliances ($Z_{N}$ and $Z_{T}$) can be defined as^31^

A1 $$ Z_{N} \equiv \mathop {\lim }\limits_{{h_{f} \to \infty }} \frac{{h_{f} }}{{\lambda_{f} + 2\mu_{f} }}, $$

and

A2 $$ Z_{T} \equiv \mathop {\lim }\limits_{{h_{f} \to \infty }} \frac{{h_{f} }}{{\mu_{f} }}, $$

where $h_{f}$ denotes the thickness fraction of fractured layer, and $\lambda_{f}$ and $\mu_{f}$ are the first and second Lamé constants of fractured layer. Then the normal and shear fracture weaknesses ($\delta_{N}$ and $\delta_{T}$) can be expressed as

A3 $$ \delta_{N} = \frac{{\left( {\lambda_{b} + 2\mu_{b} } \right)Z_{N} }}{{1 + \left( {\lambda_{b} + 2\mu_{b} } \right)Z_{N} }}, $$

and

A4 $$ \delta_{T} = \frac{{\mu_{b} Z_{T} }}{{1 + \mu_{b} Z_{T} }}, $$

where $\lambda_{b}$ and $\mu_{b}$ are the first and second Lamé constants of background rocks.

## References

[1] Nelson RA (1985). Geologic Analysis of Naturally Fractured Reservoirs.

[2] Sayers CM (2009). Seismic characterization of reservoirs containing multiple fracture sets. Geophys. Prospect..

[3] Pérez MA, Grechka V, Michelena RJ (1999). Fracture detection in a carbonate reservoir using a variety of seismic methods. Geophysics.

[4] Li, X. Fracture detection using P-P and P-S waves in multicomponent sea-floor data. in *68th SEG Annual International Meeting,**Expanded Abstracts*, 2056–2059 (1998).

[5] Jílek P (2002). Converted PS-wave reflection coefficients in weakly anisotropic media. Pure Appl. Geophys..

[6] Behura J, Tsvankin I (2006). Small-angle AVO response of PSwaves in tilted transversely isotropic media. Geophysics.

[7] Far M, Hardage B (2015). Fracture characterization using converted waves. Geophys. Prospect..

[8] Liu, E. & Martinez, A. *Seismic Fracture Characterization*. (EAGE Publications, 2013).

[9] Schoenberg M, Douma J (1998). Elastic wave propagation in media with parallel fractures and aligned cracks. Geophys. Prospect..

[10] Schoenberg M, Sayers CM (1995). Seismic anisotropy of fractured rock. Geophysics.

[11] Bakulin A, Grechka V, Tsvankin I (2000). Estimation of fracture parameters from reflection seismic data-Part I: HTI model due to a single fracture set. Geophysics.

[12] Rüger A (1997). P-wave reflection coefficients for transversely isotropic models with vertical and horizontal axis of symmetry. Geophysics.

[13] Rüger A (1998). Variation of P-wave reflectivity with offset and azimuth in anisotropic media. Geophysics.

[14] Shaw RK, Sen MK (2006). Use of AVOA data to estimate fluid indicator in a vertically fractured medium. Geophysics.

[15] Pan X, Zhang G, Yin X (2017). Azimuthally anisotropic elastic impedance inversion for fluid indicator driven by rock physics. Geophysics.

[16] Mallick S, Craft KL, Meister LJ, Chambers RE (1998). Determination of the principal directions of azimuthal anisotropy from P-wave seismic data. Geophysics.

[17] Gray, D. *Fracture Detection Using 3D Azimuthal AVO*. (CSEG Recorder, 2004).

[18] Bachrach R, Sengupta M, Salama A, Miller P (2009). Reconstruction of the layer anisotropic elastic parameter and high resolution fracture characterization from P-wave data: a case study using seismic inversion and Bayesian rock physics parameter estimation. Geophys. Prospect..

[19] Chen H, Zhang G, Ji Y, Yin X (2017). Azimuthal seismic amplitude difference inversion for fracture weakness. Pure Appl. Geophys..

[20] Far ME, Sayers CM, Thomsen L, Han DH, Castagna JP (2013). Seismic characterization of naturally fractured reservoirs using amplitude versus offset and azimuth analysis. Geophys. Prospect..

[21] Downton, J. E., & Roure, B. Interpreting azimuthal Fourier coefficients for anisotropic and fracture parameters. *Interpretation* **3**, ST9–ST27 (2015).

[22] Pratt RG (1999). Seismic waveform inversion in the frequency domain, Part 1: Theory and verification in a physical scale model. Geophysics.

[23] Buland A, Kolbjørnsen O, Omre H (2003). Rapid spatially coupled AVO inversion in the Fourier domain. Geophysics.

[24] Shin C, Min DJ (2006). Waveform inversion using a logarithmic wavefield. Geophysics.

[25] Pan X, Li L, Zhang G (2020). Multiscale frequency-domain seismic inversion for fracture weakness. J. Petrol. Sci. Eng..

[26] Thomsen L (1986). Weak elastic anisotropy. Geophysics.

[27] Downton, J. E. Seismic parameter estimation from AVO inversion: Ph.D. thesis. (University of Calgary, 2005).

[28] Zong Z, Yin X, Li K (2016). Joint AVO inversion in the time and frequency domain with Bayesian interference. Appl. Geophys..

[29] Scales, J. A., & Smith, M. L. *Introductory Geophysical Inverse Theory* (Samizdat Press, 2000).

[30] Bissantz N, Dumbgen L, Munk A, Stratmann B (2009). Convergence analysis of generalized iteratively reweighted least squares algorithms on convex function spaces. J. Optim..

[31] Brajanovski M, Gurevich B, Schoenberg M (2005). A model for P-wave attenuation and dispersion in a porous medium permeated by aligned fractures. Geophys. J. Int..

[32] Dulaijan, K. A. Inversion of azimuthal velocity and amplitude variations for seismic anisotropy. Ph. D. thesis (University of Calgary, 2017).

